# Metrics of Resident Achievement for Defining Program Aims

**DOI:** 10.5811/westjem.2021.12.53554

**Published:** 2022-01-01

**Authors:** Corlin M. Jewell, Aaron S. Kraut, Danielle T. Miller, Kaitlin A. Ray, Elizabeth Barrall Werley, Bejamin H. Schnapp

**Affiliations:** * University of Wisconsin School of Medicine and Public Health, BerbeeWalsh Department of Emergency Medicine, Madison, Wisconsin; † University of Wisconsin School of Medicine and Public Health, BerbeeWalsh Department of Emergency Medicine, Madison, Wisconsin; ‡ University of Colorado School of Medicine, Department of Emergency Medicine, Aurora, Colorado; § PennState College of Medicine, Department of Emergency Medicine, Hershey, Pennsylvania

## Abstract

**Introduction:**

Resident achievement data is a powerful but underutilized means of program evaluation, allowing programs to empirically measure whether they are meeting their program aims, facilitate refinement of curricula and improve resident recruitment efforts. The goal was to provide an overview of available metrics of resident achievement and how these metrics can be used to inform program aims.

**Methods:**

A literature search was performed using PubMed and Google Scholar between May and November of 2020. Publications were eligible for inclusion if they discussed or assessed “excellence” or “success” during residency training. A narrative review structure was chosen due to the intention to provide an examination of the literature on available resident achievement metrics.

**Results:**

57 publications met inclusion criteria and were included in the review. Metrics of excellence were grouped into larger categories, including success defined by program factors, academics, national competencies, employer factors, and possible new metrics.

**Conclusions:**

Programs can best evaluate whether they are meeting their program aims by creating a list of important resident-level metrics based on their stated goals and values using one or more of the published definitions as a foundation. Each program must define which metrics align best with their individual program aims and mission.

## INTRODUCTION

Every residency program desires to offer truly excellent training to their learners. However, excellence represents different things to different physicians, institutions, communities, and patients.

The Accreditation Council of Graduate Medical Education (ACGME) requires programs to develop aims that are reflections of the program’s mission statement as part of their Self-Study.^1^ When creating these aims, programs may benefit from “beginning with the end in mind” and considering the desired outcomes for their graduates. The identification of measurable and achievable aims targeted to an individual residency program’s unique context represents an important means of effective program evaluation and accountability. Clearly defining trainee-level aims also allows for effective program evaluation and evaluation of resident selection processes.^2^,^3^ The goal of this review is to discuss available metrics for programs to use in the creation of program aims to fit its mission.

## METHODS

### Author Group

The author group is made up of practicing U.S. emergency medicine physicians from multiple academic institutions and includes four members of the residency leadership team and two Medical Education Fellows.

### Design

A narrative design was chosen in order to examine the literature regarding possible metrics of assessing resident achievement. The scope of the review was designed to focus on actionable ideas for programs.

### Data Sources and Study Selection

Individual searches were conducted by the authors using the Google Scholar and PubMed databases for relevant keywords, including “achievement,” “success,” “resident,” “physician,” “training,” and “graduate medical education”. From the list of articles generated, a list of 17 potential metrics was generated through virtual discussion between experienced scholars using the telecommunications software Zoom (Zoom Telecommunications, Inc., San Jose) (Table 1). Other metrics of resident achievement that were less amenable to being used to create actionable program-level aims, such as being selected as chief resident, were ultimately excluded as they did not align with the purpose of this review. The 17 included metrics were used as keywords for further searches specific to each metric. Searches included articles outside the EM literature to inform program leaders of potentially underutilized metrics within the specialty. Publications were eligible for inclusion if they attempted to provide commentary on the assessment of resident performance, directly assessed resident performance, or could be potentially modified to assess resident performance during residency training using one or more of the 17 metrics. Targeted searches in the Google search engine (Mountain View, CA) using these keywords as well as review of the references section of other included manuscripts also revealed additional articles that met the inclusion criteria.

## RESULTS

Our literature search revealed 57 unique papers that met inclusion criteria for the review.

### Assessments

#### ACGME Milestones/EPAs

The ACGME outlines Milestones that provide a framework for assessing resident performance.^4^ These Milestones, along with the ACGME competencies, typically refer to abilities of the trainee. Many specialties have also created a set of Entrustable Professional Activities (EPAs) that can be used to determine the appropriate level of supervision by faculty.^5^ Resident achievement of certain Milestone levels (e.g., Level 4) could be used to ensure that a program is meeting its goals. Programs must consider what level is most appropriate to use as their standard (as achievement of particular Milestone levels is not an ACGME graduation requirement), as well as how the Milestones are assigned to ensure accuracy. Alternatively, they could determine individual Milestones they consider to be of the greatest importance and define levels for these individual areas alone.

#### Faculty Assessments

The use of faculty assessment data has been demonstrated previously to predict future success in residency.^6^ Programs could determine a certain percentage of residents achieving high aggregate numerical scores on faculty clinical assessments to be an aim suggestive of excellent clinical acumen. This approach has several advantages, including that residents are evaluated on skills that map closely to independent practice, such as creating treatment plans and working in interdisciplinary teams. However, faculty assessments can be vulnerable to bias,^7^,^8^ and faculty may not be entirely reliable evaluators of clinical performance.^9^ Further, skills such as independent learning and timely completion of administrative tasks are not well-assessed by this model, since faculty have few opportunities to observe and assess these skills. Programs could also ask for faculty gestalt of resident performance instead of using aggregate clinical assessments.^6^,^10^–^15^ While these assessments encompass all aspects of trainee performance, gestalt is ill-defined in terms of what exactly is being evaluated and concerns remain about bias.

#### Peer Assessment

Peer assessments can be another source of important feedback on resident performance. Faculty members were shown in one study to score residents higher than their peers in several sub-competency categories,^16^ including interactions that occur mainly with peers, such as transitions of care, teamwork, and communication. Creating an environment where residents are highly regarded by their peers could be an important metric to consider if a program wishes to emphasize strong personal connections between residents, professionalism, and interprofessional communication.

#### Self-Assessment

While resident self-assessment data can be useful for program evaluation, historically, learners have difficulty determining the areas in which they are deficient.^17^ However, the use of anchoring data or a framework, such as the ACGME Core Competencies and Milestones for feedback, may improve the accuracy of this self-assessment and therefore make it more useful to measure for determining success at the program level.^4^,^18^–^20^

A portfolio assembled by the resident, highlighting key examples where the resident believes they have demonstrated strength as well as weaknesses that they are working on could be used by programs in conjunction with other more traditional forms of assessment, with faculty assessing the resident’s portfolio.^21^ This may engage resident learners to reflect more extensively on their performance more than a simple self-assessment, and could be timed to align with other key interactions, such as in preparation for semi-annual or summative assessments.

### Academics

#### Fellowship Training

Fellowship training results in the attainment of specialized knowledge and skills beyond those of graduates pursuing general practice. The number of residents deciding to pursue fellowship could be a benchmark of success for programs, particularly those affiliated with academic institutions where training future leaders in the specialty is valued. This has been used previously as a means of evaluating general surgery programs.^22^

#### Academic/Administrative Leadership

The number of residents serving in leadership roles within residency programs, medical schools, or healthcare administration near the beginning of their post-residency career could represent an important focus for program evaluation.^22^ These positions represent the opportunity to create systems-level change and affect the care or education of a large number of patients or learners. However, this may be difficult to measure as these positions may not be attainable for most until several years into the postgraduate period.

#### Scholarship and Research

Residency programs have considered the number and/or quality of scholarly works produced over the course of training as a marker of excellence given that this represents one of academia’s most widely accepted currencies.^23^ Participation in research may be useful for programs attempting to boost their profile nationally or develop a more robust infrastructure for scholarship within their own department. Studies outside EM have shown that residents who participate in research during training are more likely to hold future academic positions in their field of interest than those who do not engage in research, which may be useful to programs trying to augment their profile nationally.^24^,^25^ Programs interested in this type of aim may wish to emphasize the quantity of publications, for example, or other aspects of scholarship such as presentations at regional or national conferences.

#### Examination Performance

Objective measures, such as the in-training exam (ITE) or USMLE Step 3, have been shown to correlate well with future passage of specialty board exams.^26^–^29^ However, current standardized assessments have not been shown to correlate with important markers of clinical performance, such as care provided to patients, professionalism and interpersonal communication skills.^22^,^30^ Board certification is expected by the American Board of Medical Specialties (ABMS) and is highly regarded by institutions and the general population. Passing the initial certifying exam is an important measure of excellence for continued accreditation of training programs, and a data point tracked by the ACGME.^29^ This may be most useful for programs whose residents are struggling academically or with first-time boards pass rate, who could use a score threshold as a metric for defining excellence.

#### Remediation

The need for remediation implies a deficiency in one or more ACGME core competencies, most commonly medical knowledge, patient care, and professionalism.^31^ Low or non-existent need for remediation therefore represents a potentially attractive aim to define program success. However, considering the need for remediation as a failure of the program may misinterpret residents who remediate without issue, or residents who start behind their peers but make extraordinary progress due to the appropriation of program resources as a failure rather than a success. The decision to undergo remediation is also often at the discretion of program leadership, and informal remediation typically does not involve the creation of a permanent record. Rate of remediation in EM has been measured at 4.4%, but this varies widely between specialties (<2% to >10%), suggesting this may be an unreliable metric.^31^–^34^ Tracking remediation rates could be potentially attractive to a program that has had multiple residents undergo remediation in a given year.

### Clinical

#### Performance Metrics

While clinical performance metrics hold promise as an objective measure of excellence for a residency program, it can be challenging to generate meaningful performance metrics for resident physicians that are free from significant confounders. Markers of efficient care delivery (e.g., patients per hour or number of relative value units (RVUs) generated), as well as measures of care quality (e.g., number of Emergency Department (ED) rapid return visits (i.e., “bouncebacks”) or ICU upgrades, are attractive metrics that could be defined by each program or institution. Higher resident case volumes correlated with better performance in diagnostic radiology,^35^ but no similar study exists in EM. One study showed that resident sensitive quality measures, such as the correct ordering of medications in asthma care, can be used successfully as a part of resident assessment.^36^ On the other hand, the clinical performance of resident physicians across a program is often affected by factors outside of their control, such as variance in patient acuity or their attending physicians, and may point to a need for administrative improvements or faculty development rather than any particular excellence or failure on the part of the residency program.^37^

#### Patient Satisfaction

Resident patient satisfaction scores may be another useful tool by which to benchmark program excellence. While this metric is similar to what attending physicians are measured on, it is controversial whether patient satisfaction scores are appropriate markers for quality of care received and physician performance,^38^–^40^ and it may be difficult to separate scores about the residents from perceptions of their attendings.^37^ Despite these significant limitations, training programs have begun to experiment with collecting data on resident-specific patient satisfaction scores and these could be used as one metric for defining excellence for a program.^37^

#### Procedural Competence

The successful completion of the procedures without complications is necessary for independent physician practice and is patient-centered, making this a potentially attractive target as a program aim. This is often measured by the absolute number of procedures completed through a portfolio or logbook,^41^ though the manner in which performance is assessed is variable, from direct observation gestalt to mastery learning checklists. There is some evidence, however, that the number of cases logged does not by itself demonstrate procedural competence in surgical residents, casting some doubt on its appropriateness as a program level metric.^42^

#### Adaptability

A more recent potential measure of residency program success is adaptability. With change accelerated by the COVID-19 pandemic, adaptability in an ever-changing clinical environment has become a focus, as new facets of care, such as virtual healthcare visits, are being implemented rapidly.^43^,^44^ A Master Adaptive Learner model has been proposed, suggesting that residents who develop the metacognitive skills to self-assess, self-regulate and implement new knowledge and experience may be deemed the most successful.^45^ While no data currently exists on residency programs using these skills as a measure of success, programs have been shown to play a critical role in creating a learning environment that is supportive of adaptive learning and assesses these skills.^46^

### Social

#### Community Service

The number of graduates choosing to practice in a local community may be critically important for certain residency programs. Success in this regard could encompass not only practicing medicine in these communities, but also serving in other ways, such as health literacy training.^47^ Increasing the interest of residents in serving these communities has been the subject of targeted interventions.^48^ Establishing programmatic goals and benchmarks for community service in emergency medicine may be an important way to prioritize this metric for certain programs, such as those that have poor retention of physicians in their community post-graduation.

#### Empathy

While interpersonal skills, communication, and professionalism are measured by the Core Competencies and Milestones, empathy has not previously been a target of significant performance assessment. Empathy has been rated as critically important to successful physicians and has been demonstrated to increase patient satisfaction and improve treatment outcomes.^49^ Targeted interventions at cultivating empathy have been shown to increase this quality in residents.^50^ Different instruments for measuring empathy in trainees exist and assess qualities related to empathy such as cognitive empathy (i.e. ability to recognize and understand another’s experience) and affective empathy (i.e. ability to form a bond with patients).^49^–^51^ Empathy has also been shown to decline during medical training.^52^ Therefore, the maintenance or improvement of empathy in residency as measured by these instruments despite the emotional intensity of graduate medical training could be a target for program evaluation.

#### Social Justice/Advocacy

With multiple public health crises ongoing in the U.S., such as racism, COVID-19 and gun violence, some residency training programs may consider advocacy work to be an important measure of success in their graduates. While well-studied metrics to measure the impact of these programs are currently lacking, curricula for residents have been introduced successfully into training programs.^53^,^54^ In order for EM programs to prioritize these initiatives in social justice and advocacy, potential aims could look at the number of residents involved with advocacy work, the number of successful projects introduced by trainees, or impact on the surrounding community.

#### Well-being

Well-being is an important component of success as a physician, as physician burnout has been associated with increased medical errors and decreased adherence to best practices.^55^ Programs can impact the culture of wellness among their trainees, making this an appropriate metric to consider when defining program aims. Many residency programs have implemented comprehensive curricula aimed at teaching career-long skills for maintaining well-being, as well as monitoring resident well-being throughout training.^56^,^57^ Reducing physician work hours and implementing a resident wellbeing program have been successful in reducing factors that negatively impact well-being such as emotional exhaustion.^58^,^59^ Continued measurement of wellness on a programmatic level will be important for developing wellness as a metric for residency success and could be particularly useful for programs who have undergone recent crises.

## DISCUSSION

Given the wide array of potential metrics for residency programs to define excellence, each program should thoughtfully determine which metrics are most meaningful in their environment and for the trainees that they want to produce.

Key departmental stakeholders should be included in the selection of appropriate metrics for program aims. Defining new metrics will often cause a change in how resources are deployed, so buy-in from the department chair, as well as the residents that will be directly affected by any changes, will be especially crucial. Benchmark performance levels should be set based on historical performance in the chosen area; if a residency program has never sent a graduate to fellowship, it may be unrealistic to achieve a goal of 80% of graduates choosing this option in the next few years. As the program grows and changes, the excellence metrics may need to evolve also. A program with an interest in developing a track record of resident scholarly publications for example, may want to turn its attention to academic leadership positions after several years of success.

Programs may also wish to carefully consider how their definition of excellence is used during the selection of residents to ensure they are meeting their goals while also seeking out residents who can push the program in new directions.^60^ While programs may impact the likelihood that a resident pursues scholarship or advocacy, selecting students who already have a track record of success in these areas may mean they are likely to continue to excel in this area during residency. Table 2 shows how four example residencies might use the definitions described in this manuscript to define measurable aims for themselves, using vastly different metrics depending on their local goals. It is also important to note that developed program aims can, and should, evolve over time to meet the current needs of the program and its graduates. For example, a program previously focusing on metrics associated with resident scholarship may require shifting focus to the creation of aims surrounding metrics of resident wellness in response to the COVID-19 pandemic and the emotional burden it placed on trainees.

## LIMITATIONS

This review does have limitations, many of which are inherent to the narrative design of the review. First, some relevant studies may have been missed. Given the broad range of metrics included and the lack of standardized terminology, it is difficult to ensure that all the related literature was assessed for inclusion. However, this was not designed to be an exhaustive search from its conception. Second, it is possible that there was bias in the inclusion or exclusion of certain studies given the non-systematic nature of the review. We attempted to ensure quality studies were included based on assessment by the authors who are experienced medical educators. Finally, more rigorous consensus methodology could have been employed to enhance the content validity of the review rather than the more informal discussion between the author group.

Further work will also be required to determine if the introduction of novel metrics, such as wellness or empathy scores, can be effectively used to improve outcomes via program evaluation. Additionally, future work could focus on the long-term impact of program-level metrics; for example, do physicians who initially start in an underserved community stay there, or are patient satisfaction gains sustained even after the program begins to focus in other areas.

## CONCLUSIONS

There are a variety of possible resident-level metrics which can be used for program evaluation, many of which target different aspects of performance beyond clinical skills. Each program should assess the available metrics and decide collectively on those that they consider most aligned with their program’s mission statement, aims and individual and institutional goals and use those to create measurable targets for program evaluation.

## Figures and Tables

**Table 1:** Metrics of resident success.

Assessments:
ACGME Milestones
Faculty assessment
Peer assessment
Self assessment
Academics:
Fellowship training
Academic leadership
Scholarship and Research Examination
Performance remediation
Clinical:
Clinical performance metrics
Patient satisfaction
Procedural competence
Adaptability
Social:
Community service
Empathy
Social justice/advocacy
Wellness

*ACGME*, Accreditation Council for Graduate Medical Education.

**Table 2:** Examples of incorporating metrics of resident achievement into the creation of program aims.

	Aim	Metrics	Explanation
Residency A	Train physicians with expertise in population health and an interest in serving the medically underserved.	70% of residents complete an advocacy project during residency. 50% residents continue to practice in a medically underserved area. 100% of residents are rated by faculty as “good” or “excellent” clinicians on end of 3rd year evaluations.	Residency A is committed to providing high quality care while also demonstrating a deep commitment to its social mission. By choosing metrics which reflect both, the program holds itself accountable to ensuring that neither mission is neglected.
Residency B	Train leaders in academic EM and increase the national profile of the residency program.	At least 20% of residents publish a peer reviewed manuscript during residency. 10% of residents achieve an academic leadership position within 3 years of graduation. 40% of residents choose to pursue fellowship training after graduation.	Residency B is focused on scholarly productivity and leadership development to highlight the achievements of their residents as part of a plan to develop a national reputation for their program.
Residency C	Successfully maintain accreditation.	100% of residents placed in remediation successfully complete their remediation plan. 100% of residents score in the top 50% on the In Training Exam. 100% of residents achieve at least a Level 3 across all EM Milestones.	Residency C has recently been placed on Warning status by the ACGME, so excellence in their context involves successfully preparing all residents for independent practice without incurring additional citations.
Residency D	Create a mutually supportive culture of resident wellness.	0% of residents report high levels of burnout as measured by the Maslach Burnout Inventory. 90% of residents are rated highly on teamwork by peers. 80% of residents assess themselves as “good” or “excellent” clinicians.	Residency D is interested in ensuring residents have a safe and healthy learning environment in which to grow and have prioritized metrics that focus on wellness as well as self and peer perception.
